# Partitioning Detectability Components in Populations Subject to Within-Season Temporary Emigration Using Binomial Mixture Models

**DOI:** 10.1371/journal.pone.0117216

**Published:** 2015-03-16

**Authors:** Katherine M. O’Donnell, Frank R. Thompson, Raymond D. Semlitsch

**Affiliations:** 1 Division of Biological Sciences, University of Missouri, 105 Tucker Hall, Columbia, Missouri, 65211, United States of America; 2 U.S.D.A. Forest Service Northern Research Station, Columbia, Missouri, United States of America; University of New South Wales, AUSTRALIA

## Abstract

Detectability of individual animals is highly variable and nearly always < 1; imperfect detection must be accounted for to reliably estimate population sizes and trends. Hierarchical models can simultaneously estimate abundance and effective detection probability, but there are several different mechanisms that cause variation in detectability. Neglecting temporary emigration can lead to biased population estimates because availability and conditional detection probability are confounded. In this study, we extend previous hierarchical binomial mixture models to account for multiple sources of variation in detectability. The state process of the hierarchical model describes ecological mechanisms that generate spatial and temporal patterns in abundance, while the observation model accounts for the imperfect nature of counting individuals due to temporary emigration and false absences. We illustrate our model’s potential advantages, including the allowance of temporary emigration between sampling periods, with a case study of southern red-backed salamanders *Plethodon serratus*. We fit our model and a standard binomial mixture model to counts of terrestrial salamanders surveyed at 40 sites during 3–5 surveys each spring and fall 2010–2012. Our models generated similar parameter estimates to standard binomial mixture models. Aspect was the best predictor of salamander abundance in our case study; abundance increased as aspect became more northeasterly. Increased time-since-rainfall strongly decreased salamander surface activity (i.e. availability for sampling), while higher amounts of woody cover objects and rocks increased conditional detection probability (i.e. probability of capture, given an animal is exposed to sampling). By explicitly accounting for both components of detectability, we increased congruence between our statistical modeling and our ecological understanding of the system. We stress the importance of choosing survey locations and protocols that maximize species availability and conditional detection probability to increase population parameter estimate reliability.

## Introduction

Ecologists have long recognized that population dynamics form the foundation of ecology [1,2]. Estimating how many individuals occupy various habitats is also fundamental for management and conservation. Understanding the mechanisms of population dynamics is essential for assessing the conditions of populations, predicting changes due to land use and climate change, and managing habitats in which populations live. Generating unbiased estimates of demographic parameters is crucial for such endeavors, yet parameters like abundance (*N*) are not easily measured because of imperfect detectability; detection probability (*p*) fluctuates and is nearly always < 1 [3–6]. Studies that ignore the imperfectness of the observation process may underestimate true *N* [5], report biased abundance-covariate relationships [7,8], or misidentify population trends [9] by implicitly assuming that the relationship between *p* and *N* is constant across time, space, and other factors of the study [9–11]. This assumption is rarely (if ever) true because observed counts vary spatially and temporally with changes in *p* and *N*; thus, using naïve counts for estimating *N* is precarious [3,5,8–10]. Abundance and detection probabilities must be modeled distinctly (yet simultaneously) if unbiased estimates are required [9,12].

Many population analysis methods that account for imperfect detection are labor or cost intensive (overview in [13]), but recently-developed hierarchical models allow for simultaneous estimation of population parameters and detection probability without requiring marked individuals [4,5,14–16]. A strategic benefit of the hierarchical approach is the ability to partition a complicated system into two or more simpler, linked, stochastic models that accurately represent the mechanisms generating the parameters and observations [4,5,14,16]. One such model—the binomial mixture model—was developed to estimate *N* and *p* from spatially and temporally replicated counts [16]. Extensions of the binomial mixture model have incorporated environmental covariates [17,18], correlated behavior of individuals [19], and temporal trends in open populations [3,5,20]. The model’s hierarchical structure involves: (1) a state process, which describes spatial and temporal variation in *N*, and (2) a dependent observation process that represents the filter through which we see the latent state process [16].

The observation process can be further divided into two components of detectability—availability and conditional capture probability [9,21]. Availability is determined by the presence/absence of individuals in an area, the capacity of the survey technique to detect animals of interest, and environmental factors that influence animal locations [21]. The counterpart of availability is temporary emigration, which is the probability that an individual is alive, yet unavailable to be detected during a survey [11,22]; thus, we consider temporary emigration = 1–(probability of availability for capture). Conditional capture probability is the probability that an organism is detected, given that it is available for sampling [9]. Conditional capture probability can be affected by factors such as survey methodology, observer experience level, habitat complexity, and species crypsis.

In most studies, *p* represents an overall or effective detection probability, which encompasses both availability and conditional capture probability [11,23]. However, neglecting temporary emigration can lead to biased density estimates because availability and conditional capture probability are confounded [22]. Problems with interpreting abundance estimates also occur, particularly when availability is low or varies spatiotemporally [9,21]. Many population models do not explicitly include availability, as it is not often recognized as a possible concern for many taxa [9]. Availability can be affected by the behavior or physiology or both, and by survey methods [21]. For instance, the availability of birds or frogs being counted via calls may be affected by behavioral differences between individuals or weather-related factors. Aquatic animals may be unavailable during visual surveys because they are too far below the surface or water clarity is poor. Small mammals may be unavailable for surveys due to temporary emigration into burrows, tree cavities, or other refugia. Including availability is useful in these cases, as it enables researchers to partition and model the effective detection probability in a quantitatively and biologically meaningful way.

Terrestrial woodland salamanders (family Plethodontidae) are ideal for examining the components of detectability using binomial mixture models for several reasons. First, capture-mark-recapture (CMR) is not always an option for amphibians; its labor-intensive nature means that marking enough amphibians to satisfy CMR assumptions is difficult and expensive [17,24,25]. Additionally, recapture rates are often very low for amphibians [26–28]. Second, terrestrial salamanders’ three-dimensional use of forest litter and soil is fairly unique among vertebrates. Since they lack lungs, they require moist substrate to sustain cutaneous respiration [29,30]. This high moisture requirement, coupled with terrestrial salamanders’ limited mobility, means that they exhibit limited activity on the ground surface and have small home ranges [31,32]. Terrestrial woodland salamanders often remain under surface cover objects to retain moisture, but retreat to underground burrows to prevent desiccation when surface conditions become too dry [33–35]. Therefore, unlike many other animals, terrestrial woodland salamanders’ primary direction of movement is vertical rather than horizontal, which causes high levels of daily and seasonal temporary emigration underground [11,28,36].

Terrestrial salamanders undoubtedly have ecological impacts deeper than the forest floor [28,37]; accordingly, when estimating abundance, we are interested in the total number of salamanders in an area—both at the surface and belowground. This quantity has been termed “superpopulation,” as opposed to the “surface population” consisting of salamanders available for capture [11,23,38]. As with other organisms, terrestrial salamanders’ detectability varies in two major ways: (1) spatially, because of local habitat characteristics, and (2) temporally, due to changing environmental conditions and seasonal activity patterns [11].

Our objectives were to: (1) develop a binomial mixture model that explicitly accounts for the distinct components of effective detection probability—conditional capture probability and availability and (2) compare our model to a standard binomial mixture model. For our terrestrial salamander case study, we sought to (3) identify landscape factors that best predict abundance, and (4) identify weather and habitat-related factors that best predict availability and conditional capture probability. We present our modeling approach and results of our case study using Southern red-backed salamanders *Plethodon serratus*.

## Materials and Methods

### Model development

#### State process

The state process describes the ecological mechanisms that generate spatial and temporal patterns in abundance. If sampling adheres to a metapopulation design with repeated counts of unmarked individuals (*y*_*ijk*_) occurring at *i* = 1, 2,…, *R* sites over *j* = 1, 2,…, *T* surveys (secondary periods) and *k* = 1, 2,…, *K* seasons (primary periods), then we may presume the abundance at each site (*N*_*ik*_) follows a Poisson distribution with mean *λ*_*ik*_ (eqn. 1; [5,16,20]).

$$N_{ik}|\lambda_{ik}∼\mathrm{Poisson}\left(\lambda_{ik}\right)$$ eqn 1

$$\text{log}\left(\lambda_{ik}\right)=\alpha_{\lambda \left(ik\right)}+\sum_{l=1}^{m}\beta_{\lambda \left(ikl\right)}x_{\lambda \left(ikl\right)}+\delta_{\lambda \left(ik\right)}$$ eqn 2

The parameter *λ*_*ik*_ represents the mean abundance of animals at site *i* in season *k*. We specify season-specific (*α*_*λ(k)*_) or site-by-season (*α*_*λ(ik)*_) intercepts of log *λ*. We can account for spatial heterogeneity in abundance by including *m* site and/or season-specific covariates on the log-transformed *λ*_*ik*_, as well as site-specific random effects (*δ*_*λ(ik)*_; eqn (2). We assume *N* at each site remains constant during each primary period, but abundance may change between primary periods.

#### Observation process

The observation model reflects the imperfect process of counting individuals. Repeated counts (*y*_*ijk*_) follow a binomial distribution, with index *N*_*ik*_ (per-site abundance) and success probability *p*_*ijk*_ (per-individual detection probability; eqn (3). Implicitly, *p* represents the effective detection probability, which is the product of the conditional capture probability *ω* and availability probability *ν* eqn (4). Both components of effective detection probability can vary with site, survey, and season.

$$y_{ijk}|N_{ik}∼\mathrm{Binomial}\left(N_{ik},p_{ijk}\right)$$ eqn 3

$$p_{ijk}=\nu_{ijk}\times \omega_{ijk}$$ eqn 4

We distinctly modeled the two components of *p* to more accurately reflect the separate processes that generated our observations. It is difficult to make inferences about both components of *p* without relevant explanatory variables; *ν* and *ω* remain confounded and the effective detection probability is reported [22]. However, if covariates are available that explain variation in each of the two components, then distinct parameter estimates may be identifiable. We logit-transformed *ν* and *ω* to constrain the probabilities between 0 and 1 and to incorporate covariates, which can be site, season, and/or survey-specific eqns (5, 6). Site or survey-specific random effects (*δ*) can also be included.

$$\text{logit}\left(\nu_{ijk}\right)=\alpha_{\nu \left(ijk\right)}+\sum_{l=1}^{d}\beta_{\nu \left(ijkl\right)}x_{\nu \left(ijkl\right)}+\delta_{\nu \left(ijk\right)}$$ eqn 5

$$\text{logit}\left(\omega_{ijk}\right)=\alpha_{\omega \left(ijk\right)}+\sum_{l=1}^{z}\beta_{\omega \left(ijkl\right)}x_{\omega \left(ijkl\right)}+\delta_{\omega \left(ijk\right)}$$ eqn 6

### Simulation study

To test the validity of our temporary emigration (TE) model, we evaluated its performance on simulated data for 6 different scenarios—each combination of low, moderate, and high availability intercepts (*α*_*ν*_ = 0.2, 0.5, 0.8) with moderate and high conditional capture probability intercepts (*α*_*ω*_ = 0.5, 0.9). All simulated data sets included 6 primary periods, 5 secondary periods per primary period, and 40 study sites. We simulated data using R [39] and performed analyses using JAGS [40] via the package R2jags [41]. For each simulation, we ran 3 chains for 10000 iterations, discarded the first 5000 as burn-in, and specified random starting values. We assessed convergence of all parameters using the Gelman-Rubin statistic (*R-hat* < 1.1; [42]), and conducted enough simulations to accrue 100 replicates for each scenario. We computed the bias and coverage rate (proportion of 100 posterior 95% credible intervals [CRI] that contained true parameter value) from the posterior means of *α*_*ν*_, *α*_*ω*_, *α*_*λ*_, and total abundance. R/JAGS code is included in S1 Appendix.

### Case study: Southern red-backed salamanders

*Plethodon serratus* can be found in four isolated regions in the US, including the southeastern portion of Missouri [43]. Like other terrestrial woodland salamanders, they spend much of their lives underground, but surface during favorable conditions to forage and mate. In Missouri, red-backed salamanders exhibit a seasonal activity pattern, with highest surface activity from March to May and September to October. Females oviposit during May and June, and eggs hatch between July and August [44]. These physiological constraints and life-history traits generate daily and seasonal patterns of surface activity.

**Fig 1:**
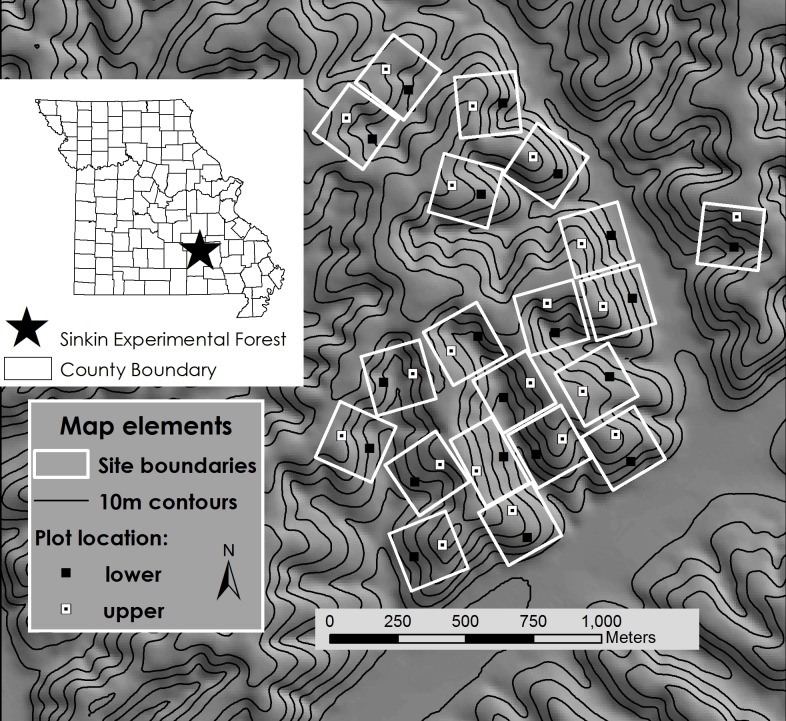
Location of study site in Dent County, Missouri, USA (inset) and relief map of 20 experimental units that were surveyed for red-backed salamanders from spring 2010—fall 2012.

We conducted surveys for *P*. *serratus* at the US Forest Service Sinkin Experimental Forest (Dent County, Missouri, USA; Fig. 1). The study site—within the Ozark Plateau—consists of mature (80–100 year old) oak and oak-pine stands (*Quercus* spp., *Pinus echinata*) that had not been harvested or thinned for ≥ 40 years [45]. We established two 10m x 10m plots within each of twenty 5-ha experimental units, yielding *i* = 40 survey plots (Fig. 1). We conducted 3–5 surveys (*j*) at all plots in each spring and fall 2010–2012 (*k* = 6 seasons); surveys lasted 2–4 days and were separated by an average (±1 SD) of 7 ± 3.7 days. We completed all surveys within each season in a short enough time span (32.1 ± 5.7 days) to assume the population was demographically closed. Terrestrial salamanders do not experience large population fluctuations over the course of a few months, so we did not expect substantial turnover or permanent emigration [17].

We conducted diurnal area-constrained searches of 3m x 3m (9-m^2^) quadrats; we searched different sections of each plot in successive surveys to avoid sampling-induced bias. Each of two observers searched 1m-wide transects by crawling through the 9-m^2^ quadrat while hand-raking leaf litter and duff and flipping natural cover objects when encountered; we continually replaced leaf litter and cover objects and ensured plots were reconstructed upon completion. Observers continued until the entire 9-m^2^ quadrat was thoroughly searched (average 9.1 ± 2.8 min); each of the 40 plots was searched in randomly determined order during each survey. For each plot, we recorded total salamanders captured, rocks (≥ 5 cm), woody cover objects (WCOs), mean soil temperature, time of day (range = 0630–1900), and mean leaf litter depth (as in [35]). We obtained rainfall and temperature data from the Sinkin Experimental Forest weather station (MSINM7). Site-specific variables of slope, Beers-transformed aspect (linear scale; southwest = 0, northeast = 2), soil water-holding capacity (AW), terrain shape index (TSI), and landform index (LFI) were determined from the Regional Oak Study [45].

We expected variation in landscape features to drive variation in abundance among sites; thus, we included aspect, slope, AW, TSI, and LFI as abundance covariates. We let the abundance intercept vary by season (*α*_*λ(k)*_; model TE[season]) and site-by-season (*α*_*λ(ik)*_; model TE[site x season]), and included a site-level random effect to account for overdispersion. For comparison, we also fit a standard binomial-mixture model (NE) that does not partition detectability. We again included a random site-level effect, let the abundance intercept vary by season (model NE[season]) and site-by-season (model NE[site x season]), and included the same covariates.

Because our survey technique targeted aboveground salamanders, we assumed availability probability *ν* was strongly associated with climatic and temporal factors that drive terrestrial salamander surface activity. Previous work suggested that time since rainfall explained over 60% of the variation among raw survey counts, which approximate salamander surface activity [35]. Thus, we included days-since-rainfall, soil temperature, time-of-day, and a quadratic time-of-day term as availability covariates. We also included a site-by-season random effect to account for unexplained variation in availability.

Conditional capture probability, by definition, is only applicable to animals that are available for capture. For our study, *ω* can be thought to represent the likelihood of an observer capturing a surface-active (i.e., available) salamander. Area-constrained searches have inherently high capture likelihoods because of their comprehensive nature and the proximity of the observer to the target organisms [46]. Thus, we assumed the intercept *α*_*ω*_ to be relatively high, and that differences in conditional capture probability among plots were primarily influenced by the structural complexity of the quadrat. Therefore, we included the covariates leaf litter depth, rocks, and WCO to reflect plot complexity.

As in the simulation study, we fit our models using JAGS [40] via the R2jags library [41] within R [39]. Prior to analysis, all covariates were standardized to promote Markov chain Monte Carlo convergence. We chose a vague normal prior for *α*_*λ*_ (mean = 0, SD = 10), weakly informative uniform priors for all coefficient terms (-3, 3) and the intercept *α*_*ν*_ (-4.6, 4.6 [0.01, 0.99 on probability scale]), and an informative normal prior for *α*_*ω*_ (mean = 2.2 [= 0.9 on probability scale], SD = 0.4). Informative priors promote model convergence by excluding unreasonably extreme values and stabilizing the logit function [4,47]. For both site-by-season abundance models, we ran 3 chains with 500000 iterations each, discarded the first 250000 as burn-in, and thinned the remaining samples by 1 in 150 to obtain 5001 samples for analysis. The season-specific abundance models required fewer iterations to achieve convergence; we ran 3 chains for 50000 iterations, discarded the initial 25000, and thinned the remainder by 1 in 15 to obtain 5001 posterior samples. We confirmed convergence using the Gelman-Rubin statistic (*R-hat* < 1.01; [42]) and assessed model fit using posterior predictive checks—we calculated a Bayesian *P*-value by comparing Chi-squared discrepancy statistics of observed to simulated data [4]. Model specification and R/JAGS code is available in S2 Appendix.

### Ethics Statement

We conducted this research in compliance with all Missouri and USA laws and regulations. The Missouri Department of Conservation approved Missouri Wildlife Collector’s Permits, and the University of Missouri Animal Care and Use Committee approved this study and its procedures (Protocol 7403).

## Results

### Simulation study

The absolute bias of *α*_*ν*_ ranged from -1 to +3% on the probability scale; coverage rate was 93–98% (Table A in S3 Appendix). The width of the 95% CRI decreased as the availability and conditional capture probabilities increased (Table A in S3 Appendix). The absolute bias of *α*_*ω*_ ranged from 0 to +3% on the probability scale; coverage rate was 91–99% (Table B in S3 Appendix). The width of the 95% CRI decreased among scenarios as the availability probability increased, but did not differ between moderate and high conditional capture probability scenarios (Table B in S3 Appendix). The mean relative bias of *α*_*λ*_ (on raw scale) was -1.5% (range: -8.5% to +1.1%); coverage rate ranged from 92–97% (Table C in S3 Appendix). The width of the 95% CRI again decreased as availability probability increased, but did not differ with conditional capture probability (Table C in S3 Appendix). The relative bias of total abundance ranged from -2.4% to +6.3% (Table D in S3 Appendix). The coverage rate for correctly estimating the abundance in all 6 seasons ranged from 72 to 94%, while the coverage rate for estimating at least 5 seasons correctly was between 90 and 97% (Table D in S3 Appendix).

### Case study: Southern red-backed salamanders

**Fig 2:**
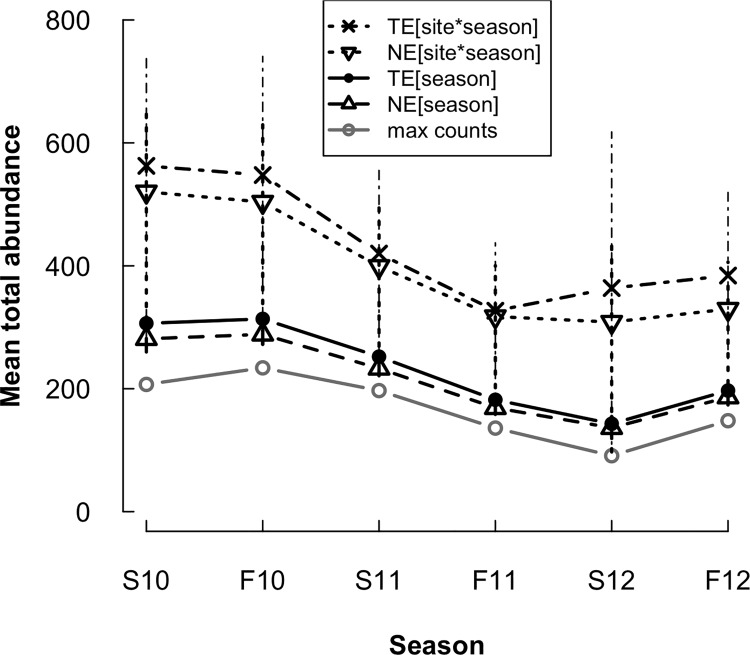
Estimates of total red-backed salamander abundance (per-season mean ± SD) from spring 2010–fall 2012. Estimated from temporary emigration models (TE) and standard binomial-mixture models (NE) versus uncorrected counts. “Max counts” = uncorrected estimates; sum of maximum counts per site per season.

We captured 2309 *P*. *serratus* during 27 sampling rounds over six seasons between 9 April 2010 and 26 October 2012. Posterior predictive checks indicated adequate fit for each of our four models (Bayesian *P*-values, fit-ratios: TE[season] = 0.338, 1.03; TE[site x season] = 0.285, 1.04; NE[season] = 0.443, 1.01; NE[site x season] = 0.373, 1.03). Estimates of per-season abundance totals differed under each of the four models (Fig. 2, Table 1). Both the TE and NE models with site-by-season abundance intercepts had higher abundance estimates than their counterparts with season-specific intercepts (Fig. 2). The mean TE[season] abundance was 53.5% of the TE[site x season] abundance; similarly, the mean NE[season] abundance was 54.5% of the NE[site x season] mean abundance. Both [site x season] models had wider 95% CRIs for all abundance-related parameters than [season] models (Tables 1 & 2). Standard deviations of site-specific random effects (abundance) and site-by-survey random effects (detection process) were significant for all models (Table 2).

**Table 1:** Per-season estimates of *P*. *serratus* abundance over 40 9-m^2^ plots.

	TE[season]		TE[site x season]		NE[season]		NE[site x season]	
	Mean (SD)	95% CRI	Mean (SD)	95% CRI	Mean (SD)	95% CRI	Mean (SD)	95% CRI
S10	306.4 (23.6)	266, 358	562.6 (177.1)	350, 1016	281.3 (19.1)	249, 324	520.4 (169.3)	321, 944
F10	313.6 (19.0)	281, 355	547.7 (192.8)	337, 1055	288.5 (14.9)	263, 321	503.7 (181.4)	318, 1000
S11	252.2 (14.7)	228, 284	419.9 (135.8)	274, 774	233.0 (11.0)	215, 258	399.2 (137.9)	260, 746
F11	182.0 (11.9)	162, 207	326.9 (110.3)	204, 606	168.9 (9.9)	153, 191	317.5 (110.4)	194, 609
S12	143.0 (13.7)	120, 173	363.8 (253.9)	167, 967	136.5 (12.5)	115, 163	308.4 (169.0)	154, 736
F12	197.0 (13.4)	175, 227	384.2 (135.0)	232, 739	186.3 (11.5)	167, 211	329.7 (99.2)	211, 564

Lower and upper values represent 95% Bayesian credible intervals. Estimated from temporary emigration (TE) and standard binomial mixture (NE) models with abundance intercepts varying by season or site-by-season.

**Table 2:** Comparison of posterior means and 95% Bayesian credible intervals of model parameters for four binomial mixture models.

		TE[season]			TE[site x season]		
	Parameter	Mean	SD	95% CRI	Mean	SD	95% CRI
Abundance	LFI	−0.057	0.121	−0.290, 0.180	−0.002	1.324	−2.529, 2.490
	TSI	0.085	0.085	−0.081, 0.255	0.181	0.951	−1.721, 2.101
	Aspect	**0.155**	**0.062**	**0.035, 0.281**	0.094	0.758	−1.428, 1.559
	AW	0.055	0.059	−0.060, 0.172	0.230	0.748	−1.248, 1.649
	Slope	0.066	0.091	−0.114, 0.249	−0.076	1.056	−2.176, 1.940
Availability	**Rain**	**−1.255**	**0.108**	**−1.476, −1.054**	**−1.076**	**0.094**	**−1.268, −0.900**
	Time	−0.151	0.090	−0.328, 0.027	−0.116	0.075	−0.262, 0.031
	Time^2^	−0.149	0.078	−0.300, 0.006	**−0.205**	**0.069**	**−0.341, −0.068**
	Temp	−0.122	0.096	−0.307, 0.071	−0.131	0.085	−0.297, 0.039
*P* | availability	Litter	0.077	0.223	−0.355, 0.516	0.117	0.275	−0.384, 0.701
	**Rocks**	**1.506**	**0.449**	**0.461, 2.228**	**1.241**	**0.505**	**0.316, 2.191**
	**WCO**	**0.474**	**0.227**	**0.135, 1.014**	**0.743**	**0.287**	**0.261, 1.343**
Random effects	**SD(site)**	**0.244**	**0.065**	**0.125, 0.378**	**0.597**	**0.447**	**0.025, 1.690**
	**SD(ν)**	**1.730**	**0.130**	**1.494, 1.995**	**1.369**	**0.117**	**1.515, 1.612**
		**NE[season]**			**NE[site x season]**		
	Parameter	Mean	SD	95% CRI	Mean	SD	95% CRI
Abundance	LFI	−0.079	0.114	−0.301, 0.144	−0.311	1.300	−2.619, 2.395
	TSI	0.100	0.081	−0.054, 0.262	0.465	0.966	−1.444, 2.170
	Aspect	**0.136**	**0.060**	**0.019, 0.255**	0.245	0.735	−1.206, 1.710
	AW	0.061	0.055	−0.046, 0.172	0.332	0.693	−0.915, 1.754
	Slope	0.089	0.084	−0.075, 0.254	0.117	1.060	−2.119, 2.085
Effective *P*	**Rain**	**−1.101**	**0.088**	**−1.279, −0.931**	**−0.985**	**0.080**	**−1.143, −0.832**
	Time	−0.121	0.074	−0.265, 0.027	−0.101	0.067	−0.232, 0.027
	**Time** ^**2**^	**−0.227**	**0.068**	**−0.359, −0.096**	**−0.255**	**0.063**	**−0.381, −0.136**
	Temp	−0.136	0.082	−0.296, 0.023	−0.139	0.075	−0.287, 0.008
	Litter	0.010	0.082	−0.149, 0.170	0.006	0.073	−0.134, 0.153
	**Rocks**	**0.424**	**0.092**	**0.245, 0.610**	**0.376**	**0.087**	**0.215, 0.547**
	**WCO**	**0.388**	**0.075**	**0.239, 0.534**	**0.330**	**0.066**	**0.203, 0.462**
Random effects	**SD(site)**	**0.218**	**0.069**	**0.079, 0.351**	**0.620**	**0.497**	**0.021, 1.852**
	**SD(*p*)**	**1.515**	**0.101**	**1.326, 1.723**	**1.277**	**0.096**	**1.098, 1.475**

NE models include effective detection probability. TE models partition effective detection probability into availability (lack of temporary emigration) and conditional detection probability. Abundance intercepts varied by season or site-by-season. Parameters with CRI not overlapping zero indicated in bold.

#### Temporary emigration models

**Fig 3:**
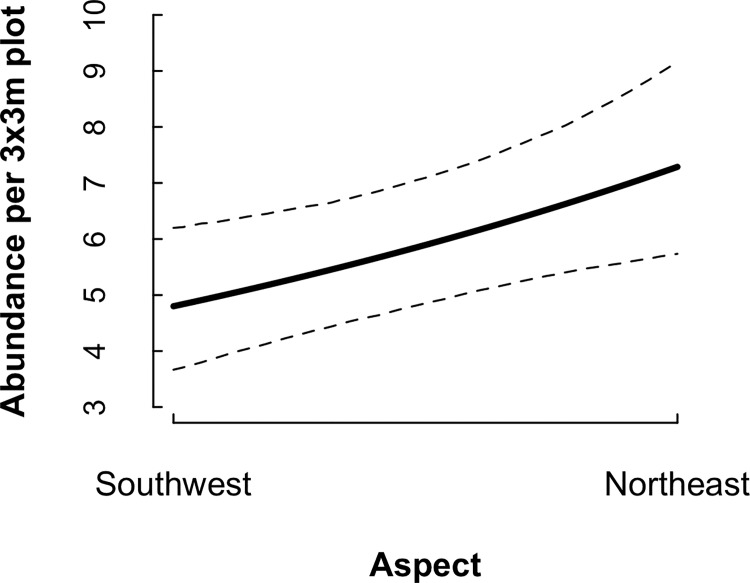
Relationship between salamander abundance and aspect in Dent County, Missouri from spring 2010–fall 2012. Predicted using model TE[season]; dashed lines represent 95% CRI.

After correcting for imperfect detection, aspect had a greater effect on salamander abundance than other landscape factors (Table 2). Abundance increased as the aspect approaches northeast, and deceased toward southwest (Fig. 3). The estimates of total abundance per season varied between temporary emigration models, but some CRIs overlapped slightly (Table 1). Fall 2010 had the highest abundance estimate under model TE[season], while Spring 2010 had the highest estimate under model TE[site x season]. Spring 2012 had the lowest abundance estimate under model TE[season], while model TE[site x season] estimated the lowest abundance in Fall 2011 (Table 1). We calculated salamander density by dividing the predicted abundance per plot by the area searched (9m^2^). Mean seasonal per-plot abundance ranged from 3.6 to 7.8 salamanders under model TE[season] and 8.2 to 14.1 under TE[site x season]; thus, mean density ranged from 0.40 to 0.87 salamanders/m^2^ under TE[season] and 0.91 to 1.57 under TE[site x season].

**Fig 4:**
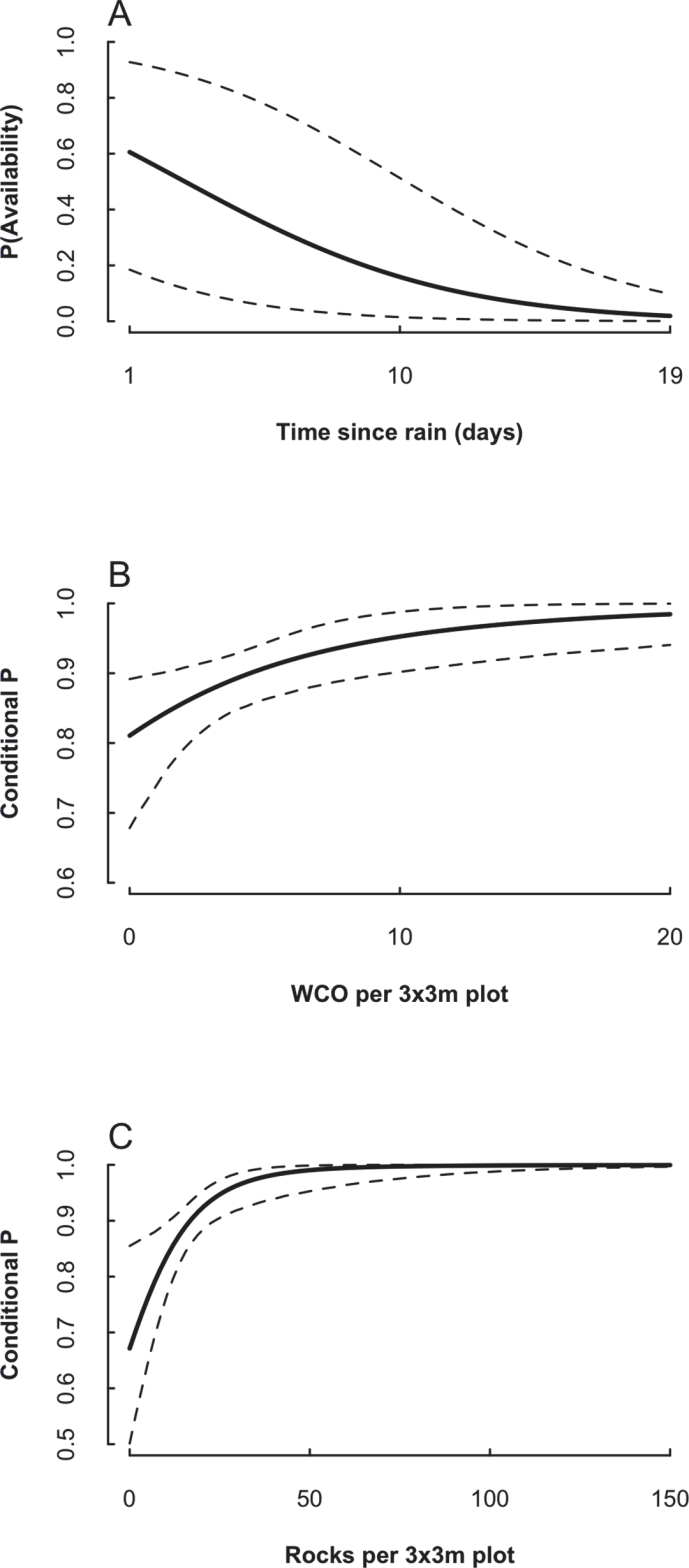
Relationships between availability probability (A), conditional detection probability (B, C), and important salamander survey covariates. Predicted using model TE[season]; dashed lines indicate 95% CRI.

**Fig 5:**
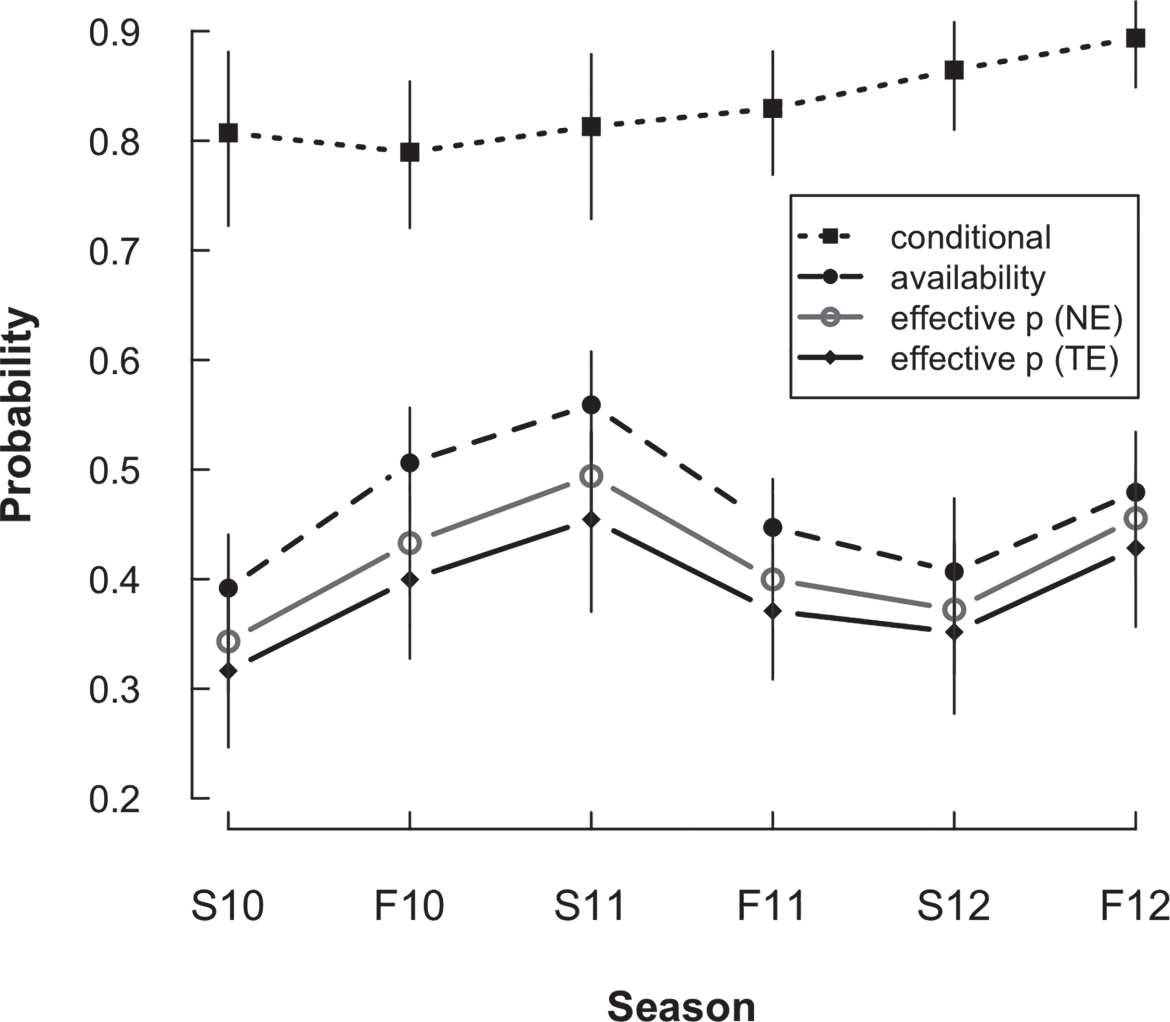
Estimates of detection probability parameters for red-backed salamander surveys (per-season mean ± SD) from spring 2010–fall 2012 using models with season-specific abundance intercepts. Conditional detection and availability probability estimates from model TE[season]. Effective detection probability (NE) estimates from model NE[season]. Effective detection probability (TE) values calculated from model TE[season].

Time-since-rainfall was the strongest predictor of salamander availability (*ν*); the CRI for the quadratic effect of time-of-day also did not overlap zero in the TE[site x season] model (Table 2). Availability steadily decreased as time-since-rainfall increased (Fig. 4A). Per-season availability averaged 0.47 (range: 0.39 to 0.56) under model TE[season] (Fig. 5) and 0.43 (range: 0.36 to 0.50) under TE[site x season]. Per-survey availability varied widely, with an overall range of 0.05 to 0.70 under TE[season] and 0.05 to 0.61 under TE[site x season] (posterior distribution plots of predicted availability in S4 Appendix).

Rock density had the greatest effect on conditional capture probability (*ω*), followed by WCO abundance (Table 2). The conditional capture probability increased as the number of WCO and rocks increased (Fig. 4B & 4C). Overall, conditional capture probability was fairly steady across seasons (Fig. 5); it averaged 0.83 under model TE[season] and 0.84 under model TE[site x season].

#### Standard binomial mixture models

Parameter estimates for both NE models were similar to their TE counterparts. Aspect had the greatest effect on abundance under model NE[season] (Table 2); salamander abundance increased as aspect approached northeast. Seasonal abundance estimates also varied between NE models, with slight overlap in CRI for a few seasons (Table 1). Fall 2010 had the highest abundance estimate under model NE[season], while Spring 2010 had the highest estimate under model NE[site x season]. Spring 2012 had the lowest abundance estimate under both NE models (Table 1). Mean seasonal per-plot salamander abundance ranged from 3.4 to 7.2 (density = 0.38 to 0.80/m^2^) under model NE[season] and 8.2 to 13.0 (density = 0.91 to 1.4/m^2^) under NE[site x season].

Time-since-rainfall had the greatest effect on effective detection probability (*p*) in both NE models (Table 2). Rocks and WCO abundance per plot had moderate positive effects on detection probability (Table 2). The quadratic of time-of-day was also important for detection probability under both models (Table 2). The mean effective detection probability per season averaged 0.42 under NE[season] and 0.39 under NE[site x season]. The per-survey detection probability was highly variable, ranging from 0.06 to 0.66 under NE[season] and 0.06 to 0.59 under NE[site x season].

## Discussion

We built an explicit description of a two-component observation process into a binomial mixture model to distinguish between two pertinent components of detectability: availability (or lack of temporary emigration) and conditional capture probability. By explicitly considering two components of the observation process, we increased congruence between our statistical model and our ecological understanding of the system. Many animals exhibit behaviors that affect their availability to be detected; examples include terrestrial mammals and invertebrates that periodically use underground burrows, aquatic animals that are not close enough to the surface to be seen, and populations in which only breeding individuals are available for capture. Our model framework is flexible, making it possible to apply to many different taxa and survey methods. Our simulation study indicates that the model is valid over a range of reasonable availability and conditional capture probability values.

Other models accounting for temporary emigration have been developed [11,22,23,38], but many involve CMR, which can be time-intensive and prohibitively expensive for amphibians [48,49] and other taxa. Chandler et al. [50] developed a single-season generalized binomial/multinomial-mixture model accounting for temporary emigration in unmarked organisms; however, their model is set in a maximum-likelihood framework, and is not open to changes in demographic parameters. Like CMR methods, temporary emigration is only allowed between primary periods, so the model cannot accommodate temporary emigration that occurs between secondary periods. In systems like ours, it makes biological sense for availability to vary among surveys (secondary periods) because terrestrial salamanders respond so strongly to changing moisture levels and temperature [35]. Our model allows for temporary emigration between secondary periods, which enables estimation of survey-specific values of availability. Like other models, it also allows fitting of site and/or season-specific covariates to both components of detection probability. Unlike the Dail-Madsen open-population binomial mixture model [51], we do not explicitly estimate between-season population dynamics (e.g., recruitment and survival rates); however, we can still observe changes in abundance estimates among seasons.

Overall, parameter estimates from our TE models did not differ greatly from corresponding NE models. The difference between posterior mean estimates from models TE[season] and NE[season] ranged from 3.5 to 38.6% for abundance covariates, 11.5 to 52.4% for availability covariates, and 18.1 to 87.0% for conditional detection covariates, however; corresponding 95% CRIs overlapped for all covariate parameter estimates. There were starker differences between models with different abundance intercept specifications (season versus site-by-season; Tables 2 & 3). This indicates that both types of models were sensitive to *α*_*λ*_ specification; the parameters from season and site-by-season models could amount to low and high estimates of salamander abundance [52]. We believe the observation that we can partition detectability into its components—and still generate similar abundance estimates to a standard binomial mixture model—is evidence of the usefulness of our model.

**Table 3:** Summary of differences between temporary emigration (TE) models and standard binomial mixture (NE) models with either season-specific or site-by-season abundance intercept.

	Abundance intercept specification	
Model type	Season	Site-by-season
**TE models**	Lower abundance than TE[site x season]	Higher abundance estimates
	More precise estimates (tighter CRI)	Less precise estimates (wider CRI)
	Partitions detectability components	Partitions detectability components
**NE models**	Lower abundance than NE[site x season]	Higher abundance estimates
	More precise estimates (tighter CRI)	Less precise estimates (wider CRI)
	Detectability not partitioned	Detectability not partitioned

We used a terrestrial salamander for our study because they are known to exhibit high levels of temporary emigration that is largely vertical, unlike many animals that wander horizontally on the landscape [11,36,49,53]. Our study further illustrated the prevalence of infrequent surface activity in terrestrial salamanders, and the importance of choosing a sampling method appropriate for the desired level of inference about a population. We estimated site and season-specific abundance, which represents the superpopulation of surface-active and belowground salamanders. We saw considerable variation in abundance among sites, but overall the most informative predictor of abundance was aspect. Highest salamander abundance is predicted on northeast slopes, while southwest slopes have the lowest predicted abundance. Northeast slopes are generally the coolest and wettest areas, which may be ideal for terrestrial salamanders that require moisture for cutaneous respiration [29,30]. Site-specific random effects on abundance encompassed overdispersion; these terms explained variation in abundance otherwise unaccounted for in the model.

We found levels of temporary emigration somewhat lower than previous studies of terrestrial salamanders that used CMR: our per-survey range was 30% to 95% (mean 47%). Buderman and Liebgold [53] found per-season temporary emigration ranged from 65% to 83%, while Bailey et al. [11,38] reported a range of 61% to 98% (mean 87%) per season. Bailey et al. [38] found that temporary emigration varied across the landscape; undisturbed/high-elevation sites had greater salamander surface activity than disturbed/low-elevation sites. They attributed the difference to decreased microhabitat variability in higher quality sites, leading to lower levels of belowground salamander emigration. In our study, salamander surface activity was primarily driven by temporally variable factors such as recent rainfall, which we used to inform the availability parameter. This allowed us to estimate a survey-specific value for availability, unlike other temporary emigration models. Variation in availability not explained by specified covariates was captured in the random survey effect.

Conditional capture probability is highly influenced by spatially variable factors such as rock and cover object density. Bailey et al. [38] reported higher conditional capture probabilities on disturbed/low-elevation sites than undisturbed/high-elevation sites. They suspected that higher conditional capture probabilities resulted from higher densities of cover objects, which may concentrate surface-active salamanders and make them easier to catch. We think that our result of conditional capture probability increasing with rock and WCO density also illustrates this point. We believe this is because the chance of capturing a salamander, given it is available, decreases as plot complexity increases; sites that have higher cover object density tend to have less vegetation, and are therefore easier to search. Search protocols also have a substantial impact on capture probability of terrestrial salamanders [53,54]. It is critical to choose methods that maximize the capture probability of available individuals; low capture probabilities often result in large confidence intervals in population parameter estimates and can make detecting population trends difficult [9,17,53].

The models we compared are designed to fit data collected in a metapopulation design—with replicate surveys over time at a number of replicate sites [16]. Previous studies have applied binomial mixture models in terrestrial salamander research [17,18,55], but none explicitly incorporated temporary emigration. Our temporary emigration model requires more information than the standard binomial-mixture model in order to partition the observation process into its two components. We collected data on spatial covariates that we believe influence conditional capture probability, and we relied on expert opinion and field experience to determine its prior distribution. In other situations, this information could be gleaned from preliminary data or a more intensive sampling regime on a subset of sites (*sensu* [10]). This ability to use pilot data or expert knowledge of a study system to set informative priors (and encourage model fitting) is a major advantage of the flexible Bayesian framework [14,20,56].

Understanding the distinction between detectability components, as well as how they are differentially affected by natural or anthropogenic disturbances, could be key in certain management decisions. Some disturbances may increase conditional detection probability by clearing survey areas and making it easier to spot organisms of interest. However, if availability is not accounted for, a false increase in effective detection probability could be perceived, leading to spurious conclusions about population estimates. For example, suppose we are interested in bird responses to wildfire, and are studying two different forest species—one green, the other brown. Before a fire, we presume the species would have similar conditional detection probabilities because they both have some camouflaging. After an intense fire that burns through the canopy, the green species would lose its camouflage and be easier for researchers to spot against the black and brown landscape. If we counted the same number of green and brown birds after the fire, but did not account for the increase in conditional capture probability of the green species, our green population estimate would be biased high, and we could miss a true population decline in the species.

Both parameters—availability and conditional detection probability—are required to fully describe the observation process that we use to make inferences about the ecological process. For robust, long-term monitoring programs, managers should select sites and survey protocols that maximize both species availability and conditional detection probability to increase precision of population parameter estimates and predictability of population trends.

## Supporting Information

S1 Appendix R/JAGS code for generation and analysis of simulated data. (DOCX)

S2 Appendix R/JAGS code for temporary emigration model. (DOCX)

S3 Appendix Simulation study description and results. (DOCX)

S4 Appendix Posterior distributions of per-survey availability. (DOCX)

## References

[1] Andrewartha H, Birch L (1954) The distribution and abundance of animals Chicago, IL: University of Chicago Press.

[2] Slobodkin LB (1980) Growth and regulation of animal populations Dover Publications.

[3] Kéry M, Royle JA (2010) Hierarchical modelling and estimation of abundance and population trends in metapopulation designs. J Anim Ecol 79: 453–461. 10.1111/j.1365-2656.2009.01632.x 19886893

[4] Kéry M, Schaub M (2012) Bayesian Population Analysis using WinBUGS: A Hierarchical Perspective New York: Academic Press.

[5] Royle JA, Dorazio RM (2008) Hierarchical Modeling and Inference in Ecology 1st ed. New York: Academic Press.

[6] Royle JA, Kéry M, Gautier R, Schmid H (2007) Hierarchical spatial models of abundance and occurrence from imperfect survey data. Ecol Monogr 77: 465–481.

[7] Tyre A, Tenhumberg B, Field S (2003) Improving precision and reducing bias in biological surveys: estimating false-negative error rates. Ecol Appl 13: 1790–1801.

[8] Kéry M (2008) Estimating abundance from bird counts: Binomial mixture models uncover complex covariate relationships. Auk 125: 336–345.

[9] Kéry M, Schmidt B (2008) Imperfect detection and its consequences for monitoring for conservation. Community Ecol 9: 207–216.

[10] Pollock KH, Nichols JD, Simons TR, Farnsworth GL, Bailey LL, et al (2002) Large scale wildlife monitoring studies: statistical methods for design and analysis. Environmetrics 13: 105–119.

[11] Bailey LL, Simons TR, Pollock KH (2004) Estimating detection probability parameters for *Plethodon* salamanders using the robust capture-recapture design. J Wildl Manage 68: 1–13.

[12] Mackenzie DI, Kendall WL (2002) How should detection probability be incorporated into estimates of relative abundance? Ecology 83: 2387–2393.

[13] Williams BK, Nichols JD, Conroy MJ (2002) Analysis and Management of Animal Populations: Modeling, Estimation, and Decision Making San Diego: Academic Press.

[14] Halstead BJ, Wylie GD, Coates PS, Valcarcel P, Casazza ML (2012) “Exciting statistics”: the rapid development and promising future of hierarchical models for population ecology. Anim Conserv 15: 133–135.

[15] Mackenzie DI, Nichols JD, Lachman G (2002) Estimating site occupancy rates when detection probabilities are less than one. Ecology 83: 2248–2255.

[16] Royle JA (2004) N-mixture models for estimating population size from spatially replicated counts. Biometrics 60: 108–115. 15032780 10.1111/j.0006-341X.2004.00142.x

[17] Dodd CK Jr., Dorazio RM (2004) Using counts to simultaneously estimate abundance and detection probabilities in a salamander community. Herpetologica 60: 468–478.

[18] Peterman WE, Semlitsch RD (2013) Fine-scale habitat associations of a terrestrial salamander: the role of environmental gradients and implications for population dynamics. PLoS One 8: e62184 10.1371/journal.pone.0062184 23671586 PMC3646024

[19] Martin J, Royle JA, Mackenzie DI, Edwards HH, Kéry M, et al (2011) Accounting for non-independent detection when estimating abundance of organisms with a Bayesian approach. Methods Ecol Evol 2: 595–601.

[20] Kéry M, Dorazio RM, Soldaat L, van Strien A, Zuiderwijk A, et al (2009) Trend estimation in populations with imperfect detection. J Appl Ecol 46: 1163–1172.

[21] Pollock KH, Marsh H, Bailey LL, Alldredge MW (2004) Separating Components of Detection Probability in Abundance Estimation : An Overview with Diverse Examples In: Thompson WL, editor. Sampling Rare or Elusive Species: Concepts, Designs, and Techniques for Estimating Population Parameters. Island Press pp. 43–58.

[22] Kendall WL, Nichols JD, Hines JE (1997) Estimating temporary emigration using capture-recapture data with Pollock’s robust design. Ecology 78: 563–578.

[23] Kendall WL (1999) Robustness of closed capture-recapture methods to violations of the closure assumption. Ecology 80: 2517–2525.

[24] Donnelly MA, Guyer C (1994) Mark-recapture In: Heyer W, Donnelly M, McDiarmid R, Heyer L, Foster MS, editors. Measuring and Monitoring Biological Diversity. Standard Methods for Amphibians. Smithsonian Institution Press pp. 183–200.

[25] Pollock KH, Nichols JD, Brownie C, Hines JE (1990) Statistical inference for capture-recapture experiments. Wildl Monogr 107: 1–97.

[26] Jung RE, Droege S, Sauer JR, Landy RB (2000) Evaluation of terrestrial and streamside salamander monitoring techniques at Shenandoah National Park. Environ Monit Assess 63: 65–79.

[27] Smith CK, Petranka JW (2000) Monitoring terrestrial salamanders: repeatability and validity of area-constrained cover object searches. J Herpetol 34: 547–557.

[28] Taub F (1961) The distribution of the red-backed salamander, *Plethodon c*. *cinereus*, within the soil. Ecology 42: 681–698.

[29] Spotila JR (1972) Role of temperature and water in the ecology of lungless salamanders. Ecol Monogr 42: 95–125.

[30] Feder ME (1983) Integrating the ecology and physiology of plethodontid salamanders. Herpetologica 39: 291–310.

[31] Liebgold EB, Brodie ED, Cabe PR (2011) Female philopatry and male-biased dispersal in a direct-developing salamander, *Plethodon cinereus* . Mol Ecol 20: 249–257. 10.1111/j.1365-294X.2010.04946.x 21134012

[32] Kleeberger S, Werner J (1982) Home range and homing behavior of *Plethodon cinereus* in northern Michigan. Copeia 1982: 409–415.

[33] Jaeger RG (1980) Microhabitats of a terrestrial forest salamander. Copeia 1980: 265–268.

[34] Grover MC (1998) Influence of cover and moisture on abundances of the terrestrial salamanders Plethodon cinereus and Plethodon glutinosus. J Herpetol 32: 489–497.

[35] O’Donnell KM, Thompson FR III, Semlitsch RD (2014) Predicting variation in microhabitat utilization of terrestrial salamanders. Herpetologica 70: 259–265.

[36] Price SJ, Eskew EA, Cecala KK, Browne RA, Dorcas ME (2012) Estimating survival of a streamside salamander: Importance of temporary emigration, capture response, and location. Hydrobiologia 679: 205–215.

[37] Davic RD, Welsh HH (2004) On the ecological roles of salamanders. Annu Rev Ecol Evol Syst 35: 405–434.

[38] Bailey LL, Simons TR, Pollock KH (2004) Spatial and temporal variation in detection probabilty of *Plethodon* salamanders using the robust capture-recapture design. J Wildl Manage 68: 14–24.

[39] R Core Team (2013) R: A language and environment for statistical computing. Available: http://www.r-project.org/.

[40] Plummer M (2003) JAGS: A program for analysis of Bayesian graphical models using Gibbs sampling.

[41] Su Y-S, Yajima M (2013) R2jags: A package for running jags from R. Available: http://cran.r-project.org/package=R2jags.

[42] Gelman A, Hill J (2007) Data analysis using regression and multilevel/hierarchical models 1st ed. New York: Cambridge University Press.

[43] Petranka JW (1998) Salamanders of the United States and Canada Washington DC: Smithsonian Institution Press.

[44] Herbeck LA, Semlitsch RD (2000) Life history and ecology of the southern redback salamander, Plethodon serratus, in Missouri. J Herpetol 34: 341–347.

[45] Kabrick JM, Villwock JL, Dey DC, Keyser TL, Larsen DR (2014) Modeling and mapping oak advance reproduction density using soil and site variables. For Sci 60.

[46] Jaeger RG, Inger RF (1994) Quadrat sampling In: Heyer WR, Donnelly MA, McDiarmid RW, Hayek LC, Foster MS, editors. Measuring and Monitoring Biological Diversity: Standard Methods for Amphibians. Washington DC: Smithsonian Institution Press pp. 97–102.

[47] Gelman A, Jakulin A, Pittau MG, Su Y-S (2008) A weakly informative default prior distribution for logistic and other regression models. Ann Appl Stat 2: 1360–1383.

[48] Dodd CK Jr. (2003) Monitoring amphibians in Great Smoky Mountains National Park U.S. Geological Survey.

[49] Mazerolle MJ, Bailey LL, Kendall WL, Royle JA, Converse SJ, et al (2007) Making great leaps forward: Accounting for detectability in herpetological field studies. J Herpetol 41: 672–689. 17328165

[50] Chandler RB, Royle JA, King D (2011) Inference about density and temporary emigration in unmarked populations. Ecology 92: 1429–1435. 21870617 10.1890/10-2433.1

[51] Dail D, Madsen L (2011) Models for estimating abundance from repeated counts of an open metapopulation. Biometrics 67: 577–587. 10.1111/j.1541-0420.2010.01465.x 20662829

[52] Semlitsch RD, O’Donnell KM, Thompson FR (2014) Abundance, biomass production, nutrient content, and the possible role of terrestrial salamanders in Missouri Ozark forest ecosystems. Can J Zool. Available: 10.1139/cjz-2014-0141.

[53] Buderman FE, Liebgold EB (2012) Effect of search method and age class on mark-recapture parameter estimation in a population of red-backed salamanders. Popul Ecol 54: 157–167.

[54] Williams AK, Berkson J (2004) Reducing false absences in survey data: Detection probabilities of red-backed salamanders. J Wildl Manage 68: 418–428.

[55] McKenny HC, Keeton WS, Donovan TM (2006) Effects of structural complexity enhancement on eastern red-backed salamander (*Plethodon cinereus*) populations in northern hardwood forests. For Ecol Manage 230: 186–196.

[56] Bolker B, Gardner B, Maunder M, Berg C, Brooks M, et al (2013) Strategies for fitting nonlinear ecological models in R, AD Model Builder, and BUGS. Methods Ecol Evol 4: 501–512.

