# Supplementary material for: Partitioning Detectability Components in Populations Subject to Within-Season Temporary Emigration Using Binomial Mixture Models
Source: PLoS One. 2015 Mar 16;10(3):e0117216. doi: 10.1371/journal.pone.0117216 (PMC4361623; doi:10.1371/journal.pone.0117216)
Supplement: S1 Appendix — (DOCX) [file pone.0117216.s001.docx]

**S1 APPENDIX.** R/JAGS code for generation and analysis of simulated data

###########################################

#### function for generating data in R ####

###########################################

data.fn <- function(R=40, T=5, K=6, #sites, surveys, seasons

x1mean=2, #covariate of detection

x2min=-1.5, x2max=1.5, #abundance covariate

x3n=T*K, x3mean=2, #covariate of availability

Talpha.om=2.2, #true values of parameters

Talpha.nu=-1.39, #alpha.om and alpha.nu vary by scenario

Talpha.lam=1.5,

Tbeta.om=1,

Tbeta.lam=0.8,

Tbeta.nu=-0.5){

y <- array(dim=c(R,T,K)) #array for counts

N.tr <- array(dim=c(R,K)) #array for true abundance per site per season

#### ecological process ####

X2 <- (runif(n=R, min=x2min, max=x2max)) #create site-level covariate

lam.tr <- exp(Talpha.lam + Tbeta.lam*X2) #abundance-covariate relationship

for (k in 1:K){

N.tr[,k] <- rpois(n=R, lambda=lam.tr) #add Poisson variation

}

#### observation process ####

X3 <- array((rep((rpois(x3n,x3mean)),each=R)), dim=c(R,T,K)) #survey covariate

nu.tr <- plogis(Talpha.nu + Tbeta.nu*X3) #availability-covariate relationship

X1 <- array((rpois(n=R*T*K, x1mean)), dim=c(R,T,K)) #site*survey covariate om.tr <- plogis(Talpha.om + Tbeta.om*X1) #conditional p-covariate relationship

p.tr <- nu.tr*om.tr #overall detection probability

#### count process ####

for (i in 1:R){

for (k in 1:K){

for (j in 1:T){

y[i,j,k] <- rbinom(1, size=N.tr[i,k], prob=p.tr[i,j,k])

}}}

#### return data ####

return(list(R=R, T=T, X1=X1, X2=X2, X3=X3, y=y, N.tr=N.tr))

} #end data function

####################################################################

#### TE binomial mixture model #### run in R, requires “R2jags” ####

####################################################################

sink("TEsim.txt")

cat("

model {

## priors

alpha.om ~ dunif(-4.6,4.6) #equates to (.01, .99) on probability scale

alpha.nu ~ dunif(-4.6,4.6) #to prevent numerical overflow/underflow

alpha.lam ~ dunif(-5,5)

beta.om ~ dunif(-5,5)

beta.lam ~ dunif(-5,5)

beta.nu ~ dunif(-5,5)

## ecological model

for (k in 1:6){

for (i in 1:R){

N[i,k] ~ dpois(lambda[i,k])

log(lambda[i,k]) <- alpha.lam + beta.lam*X2[i]

## observation model

for (j in 1:T){

y[i,j,k] ~ dbin(p[i,j,k],N[i,k])

p[i,j,k] <- (om[i,j,k])*(nu[i,j,k])

om[i,j,k] <- exp(logit.om[i,j,k])/(1+exp(logit.om[i,j,k]))

logit.om[i,j,k] <- alpha.om + beta.om*X1[i,j,k]

nu[i,j,k] <- exp(logit.nu[i,j,k])/(1+exp(logit.nu[i,j,k]))

logit.nu[i,j,k] <- alpha.nu + beta.nu*X3[i,j,k]

} #close j loop

} #close i loop

} #close k loop

# Derived quantities

for (k in 1:6){

totalN[k] <- sum(N[,k]) #total population for each season (across all sites)

}

} #close model loop

",fill=TRUE)

sink()

##initial values

Ninit <- apply(y, c(1,3), max) + 1 #max for each site and season +1 (so no 0’s)

inits <- function() list(N=Ninit, alpha.lam=runif(1,-1, 1), alpha.om=runif(1,-1, 1), alpha.nu=runif(1,-1, 1), beta.lam=runif(1,-1,1), beta.om=runif(1,-1, 1), beta.nu = runif(1,-1, 1))

##parameters monitored

params <- c("alpha.lam", "alpha.om", "alpha.nu", "beta.nu", "beta.om", "beta.lam", "N", "totalN")

#MCMC settings

ni <- 10000

nt <- 2

nb <- 5000

nc <- 3

#call JAGS for each simulation

data <- data.fn()

out <- jags(data, inits, params, "TEsim.txt", n.chains = nc, n.thin = nt,

n.iter = ni, n.burnin = nb, working.directory = getwd())
