# Supplementary material for: Partitioning Detectability Components in Populations Subject to Within-Season Temporary Emigration Using Binomial Mixture Models
Source: PLoS One. 2015 Mar 16;10(3):e0117216. doi: 10.1371/journal.pone.0117216 (PMC4361623; doi:10.1371/journal.pone.0117216)
Supplement: S2 Appendix — (DOCX) [file pone.0117216.s002.docx]

**S2 APPENDIX.** R/JAGS code for temporary emigration model

model {

# Priors

for (k in 1:6){

alpha.om ~ dnorm(2.2,6) #conditional detection probability

for (i in 1:R){

alpha.lam[i,k] ~ dnorm(0, 0.01) #for model TE[k], alpha.lam[k]

alpha.nu[i,k] ~ dunif(-4.6, 4.6) #same as (0.01, 0.99) on prob scale

}

}

for (i in 1:R){

lam.site[i] ~ dnorm(0,tau.site)T(-20,20) #random site effect

}

tau.nu <- 1 / (sd.nu * sd.nu) #random availability effect

sd.nu ~ dunif(0,5)

tau.site<-1/(sd.site*sd.site)

sd.site~dunif(0,5)

beta.aspect ~ dunif(-3,3)

beta.slope ~ dunif(-3,3)

beta.aw ~ dunif(-3,3)

beta.TSI ~ dunif(-3,3)

beta.LFI ~ dunif(-3,3)

beta.nu.r ~ dunif(-3,3) #rain

beta.nu.ti ~ dunif(-3,3) #time of day

beta.nu.te ~ dunif(-3,3) #soil temperature

beta.nu.t2 ~ dunif(-3,3) #(time of day)^2

beta.om.rk ~ dunif(-3,3) #rocks

beta.om.w ~ dunif(-3,3) #woody cover

beta.om.ll ~ dunif(-3,3) #leaf litter

# State process model for abundance

for (i in 1:R){ # Loop over R sites (40)

for (k in 1:6){ # Loop over k seasons (6)

log(lambda[i,k]) <- alpha.lam[i,k] + beta.aspect*aspect[i] + beta.slope*slope[i] + beta.aw*aw[i] + beta.TSI*TSI[i] + beta.LFI*LFI[i] + beta.lfm*lfm[i] + lam.site[i]

N[i,k] ~ dpois(lambda[i,k])

# Observation model

for (j in 1:Tr[k]){ # Loop over surveys (3-5)

y[i,j,k] ~ dbin(p[i,j,k], N[i,k])

p[i,j,k] <- (nu[i,j,k])*(om[i,j,k])

#availability

nu[i,j,k] <- exp(logit.nu[i,j,k])/(1+exp(logit.nu[i,j,k]))

logit.nu[i,j,k] ~ dnorm(mu.ln[i,j,k], tau.nu)

mu.ln[i,j,k] <- alpha.nu[i,k] + beta.nu.r*rain[i,j,k] + beta.nu.ti*time[i,j,k] + beta.nu.te*temp[i,j,k] + beta.nu.t2*time2[i,j,k]

#conditional det prob

om[i,j,k] <- exp(logit.om[i,j,k])/(1+exp(logit.om[i,j,k]))

logit.om[i,j,k] <- alpha.om[k] + beta.om.rk*rocks[i,j,k] + beta.om.ll*litter[i,j,k] + beta.om.w*wco[i,j,k]

# Assess model fit adequacy using Chi-squared discrepancy (for Bayesian P)

# Compute fit statistic FIT for observed data

expect[i,j,k] <- p[i,j,k]*N[i,k] # Expected values

FIT[i,j,k] <- pow((y[i,j,k] - expect[i,j,k]),2)/(expect[i,j,k] + 0.5)

# Generate replicate data and compute fit stats for them

y.new[i,j,k] ~ dbin(p[i,j,k], N[i,k])

FIT.new[i,j,k] <- pow((y.new[i,j,k] - expect[i,j,k]),2)/(expect[i,j,k] + 0.5)

} #j

} #i

} #k

# Derived and other quantities

for (k in 1:6){

totalN[k] <- sum(N[,k]) # Total pop. size across all sites

mean.abundance[k] <- mean(lambda[,k])

}

fit <- (sum(FIT[ ,1:5,1:4])) + (sum(FIT[ ,1:3,5])) + (sum(FIT[ ,1:4,6]))

fit.new <- (sum(FIT.new[ ,1:5,1:4])) + (sum(FIT.new[ ,1:3,5])) + (sum(FIT.new[ ,1:4,6])

}
