# Supplementary material for: Partitioning Detectability Components in Populations Subject to Within-Season Temporary Emigration Using Binomial Mixture Models
Source: PLoS One. 2015 Mar 16;10(3):e0117216. doi: 10.1371/journal.pone.0117216 (PMC4361623; doi:10.1371/journal.pone.0117216)
Supplement: S3 Appendix — (DOCX) [file pone.0117216.s003.docx]

**S3 APPENDIX.** **Simulation study description and results**

We conducted a simulation study to evaluate the temporary emigration model over a range of availability and conditional capture probabilities. We simulated data using the function in S1 Appendix in program R under 6 scenarios: all combinations of low, moderate, and high availability intercepts (*α_ν_* = 0.2, 0.5, 0.8) with both moderate and high conditional capture probability intercepts (*α_ω_* = 0.5, 0.9). For all scenarios, we generated data sets of observed counts over 6 seasons with 5 surveys per season at 40 study sites. We included a site-level covariate on abundance, a survey-specific covariate on availability, and a site-by-survey covariate on conditional capture probability. Covariate values were randomly generated for each simulation, but were all based on the same parameter values. The values of covariate effects (slope parameters) were also identical for all simulations.

We analyzed data under the temporary emigration model using JAGS via the package R2jags. For each simulation, we ran 3 chains for 10000 iterations and discarded the first 5000 as burn-in. We specified random starting values from the prior distributions of each parameter. We ran simulations under each scenario until we accumulated 100 converged model runs, assessed using the Gelman-Rubin statistic (R-hat < 1.1; Gelman & Hill 2007). Full results from simulation study are summarized in the tables below.

**Table A.** Posterior mean estimates and 95% Bayesian credible intervals of *alpha.nu* (availability probability intercept) from 100 simulated data sets. Coverage rate indicates the frequency with which the true parameter value was within the posterior 95% credible interval. Data were simulated under the temporary emigration (TE) model for 6 scenarios. Parameters common to all data sets: alpha.lam=1.5; beta.om=1; beta.lam=0.8; beta.nu= –0.5; R=40 sites; K=6 seasons; Tr=5 surveys per season. *α_nu_* = availability probability intercept, *α_omega_* = conditional capture probability intercept.

|  |  |  | *α_omega_* = 0.5 | |  | *α_omega_* = 0.9 | |
| --- | --- | --- | --- | --- | --- | --- | --- |
|  | **Scale** | **Truth** | **Mean (95% CRI)** | **Coverage** |  | **Mean (95% CRI)** | **Coverage** |
| *α_nu_* = 0.2 | Probability | 0.20 | 0.23 (0.12, 0.39) | 0.93 |  | 0.21 (0.12, 0.33) | 0.98 |
|  | Logit | -1.39 | -1.22 (-1.96, -0.46) |  |  | -1.35 (-2.04, -0.71) |  |
| *α_nu_* = 0.5 | Probability | 0.50 | 0.49 (0.39, 0.59) | 0.94 |  | 0.50 (0.42, 0.58) | 0.97 |
|  | Logit | 0.00 | -0.05 (-0.45, 0.35) |  |  | 0.01 (-0.31, 0.33) |  |
| *α_nu_* = 0.8 | Probability | 0.80 | 0.80 (0.75, 0.85) | 0.95 |  | 0.79 (0.76, 0.83) | 0.96 |
|  | Logit | 1.39 | 1.40 (1.08, 1.75) |  |  | 1.35 (1.14, 1.59) |  |

**Table B.** Posterior mean estimates and 95% Bayesian credible intervals of *alpha.om* (conditional capture probability intercept) from 100 simulated data sets. Coverage rate indicates the frequency with which the true parameter value was within the posterior 95% credible interval. Data were simulated under the temporary emigration (TE) model for 6 scenarios. Parameters common to all data sets: alpha.lam=1.5; beta.om=1; beta.lam=0.8; beta.nu= –0.5; R=40 sites; K=6 seasons; Tr=5 surveys per season. *α_nu_* = availability probability intercept, *α_omega_* = conditional capture probability intercept.

|  |  |  | *α_omega_* = 0.5 | |  |  | *α_omega_* = 0.9 | |
| --- | --- | --- | --- | --- | --- | --- | --- | --- |
|  | **Scale** | **Truth** | **Mean (95% CRI)** | **Coverage** |  | **Truth** | **Mean (95% CRI)** | **Coverage** |
| *α_nu_* = 0.2 | Probability | 0.5 | 0.50 (0.32, 0.72) | 0.93 |  | 0.9 | 0.92 (0.67, 0.99) | 0.99 |
|  | Logit | 0.0 | 0.00 (-0.74, 0.95) |  |  | 2.2 | 2.51 (0.70, 4.38) |  |
| *α_nu_* = 0.5 | Probability | 0.5 | 0.50 (0.42, 0.59) | 0.93 |  | 0.9 | 0.92 (0.79, 0.98) | 0.97 |
|  | Logit | 0.0 | 0.00 (-0.33, 0.38) |  |  | 2.2 | 2.50 (1.30, 4.07) |  |
| *α_nu_* = 0.8 | Probability | 0.5 | 0.50 (0.45, 0.55) | 0.99 |  | 0.9 | 0.93 (0.86, 0.98) | 0.91 |
|  | Logit | 0.0 | -0.01 (-0.21, 0.19) |  |  | 2.2 | 2.59 (1.78, 3.79) |  |

**Table C.** Posterior mean estimates and 95% Bayesian credible intervals of *alpha.lam* (abundance intercept) from 100 simulated data sets. Coverage rate indicates the frequency with which the true parameter value was within the posterior 95% credible interval. Data were simulated under the temporary emigration (TE) model for 6 scenarios. Parameters common to all data sets: alpha.lam=1.5; beta.om=1; beta.lam=0.8; beta.nu= –0.5; R=40 sites; K=6 seasons; Tr=5 surveys per season. *α_nu_* = availability probability intercept, *α_omega_* = conditional capture probability intercept.

|  |  |  | *α_omega_* = 0.5 | |  | *α_omega_* = 0.9 | |
| --- | --- | --- | --- | --- | --- | --- | --- |
|  | **Scale** | **Truth** | **Mean (95% CRI)** | **Coverage** |  | **Mean (95% CRI)** | **Coverage** |
| *α_nu_* = 0.2 | Raw | 4.48 | 4.10 (2.59, 7.17) | 0.92 |  | 4.44 (2.89, 7.85) | 0.97 |
|  | Log | 1.50 | 1.40 (0.95, 1.97) |  |  | 1.49 (1.06, 2.06) |  |
| *α_nu_* = 0.5 | Raw | 4.48 | 4.53 (3.74, 5.58) | 0.93 |  | 4.44 (3.82, 5.31) | 0.95 |
|  | Log | 1.50 | 1.51 (1.32, 1.72) |  |  | 1.49 (1.34, 1.67) |  |
| *α_nu_* = 0.8 | Raw | 4.48 | 4.48 (4.10, 4.90) | 0.96 |  | 4.48 (4.14, 4.85) | 0.97 |
|  | Log | 1.50 | 1.50 (1.41, 1.59) |  |  | 1.50 (1.42, 1.58) |  |

**Table D.** Coverage rate and relative bias for total abundance estimates from 100 simulated data sets. Coverage indicates proportion of posterior 95% credible intervals for estimated abundance that contained true total abundance value. Relative bias calculated as mean discrepancy of all 6 seasons. Data were simulated under the temporary emigration (TE) model for 6 scenarios. Parameters common to all data sets: alpha.lam=1.5; beta.om=1; beta.lam=0.8; beta.nu= –0.5; R=40 sites; K=6 seasons; Tr=5 surveys per season. *α_nu_* = availability probability intercept, *α_omega_* = conditional capture probability intercept.

|  | *α_omega_* = 0.5 | | |  | *α_omega_* = 0.9 | | |
| --- | --- | --- | --- | --- | --- | --- | --- |
|  | **Relative bias** | **Coverage rate** | |  | **Relative bias** | **Coverage rate** | |
|  |  | ≥ 5 seasons | 6 seasons |  |  | ≥ 5 seasons | 6 seasons |
| *α_nu_* = 0.2 | -0.024 | 0.92 | 0.89 |  | 0.063 | 0.97 | 0.94 |
| *α_nu_* = 0.5 | 0.028 | 0.92 | 0.85 |  | 0.0054 | 0.92 | 0.83 |
| *α_nu_* = 0.8 | 0.0050 | 0.92 | 0.72 |  | -0.0005 | 0.90 | 0.78 |
