# Supplementary material for: Partitioning Detectability Components in Populations Subject to Within-Season Temporary Emigration Using Binomial Mixture Models
Source: PLoS One. 2015 Mar 16;10(3):e0117216. doi: 10.1371/journal.pone.0117216 (PMC4361623; doi:10.1371/journal.pone.0117216)

**S4 Appendix. Posterior distributions of per-survey availability**. Calculated using model TE[season] and shown on predicted (probability) scale. Indices represent [season #, survey #].


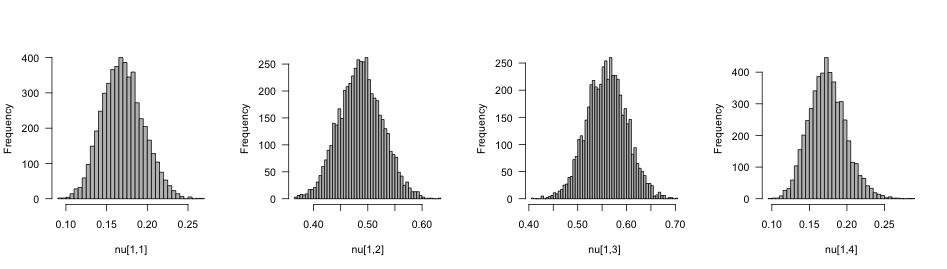


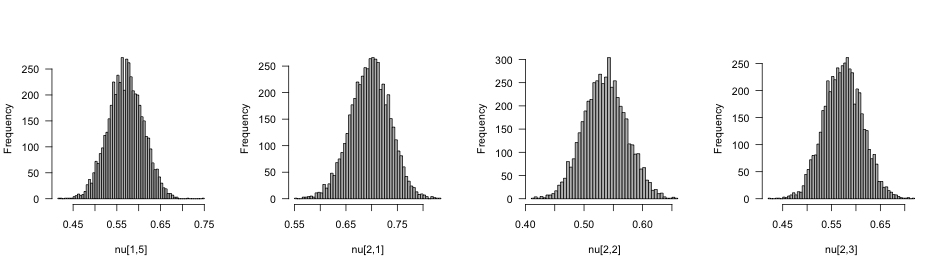


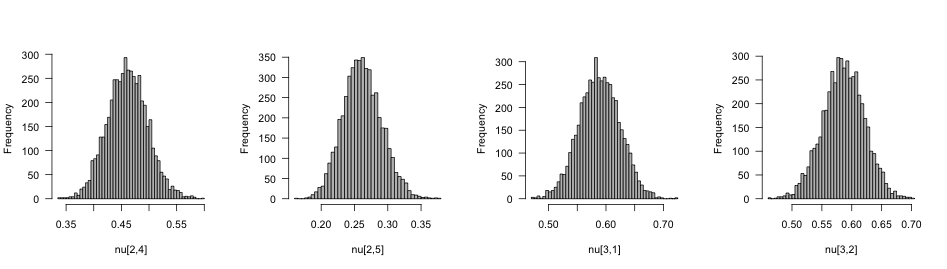


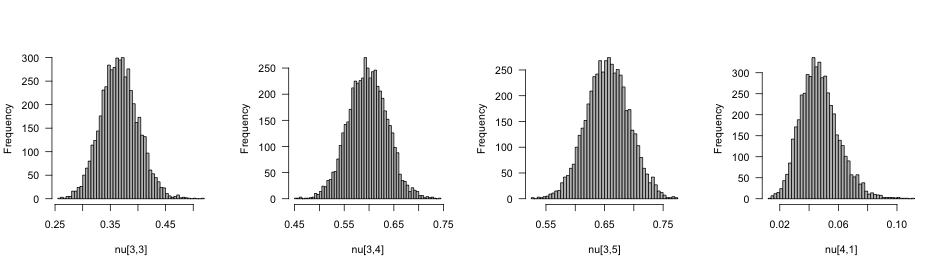


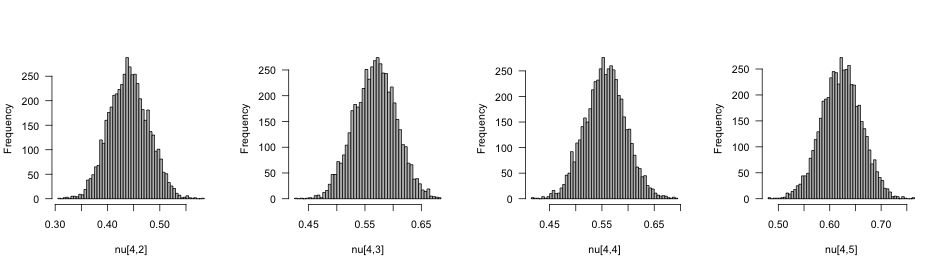


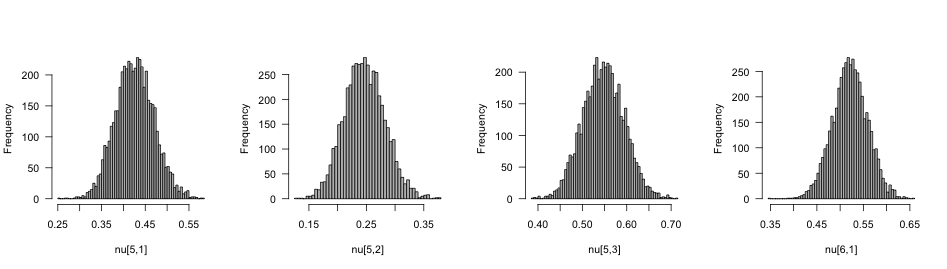


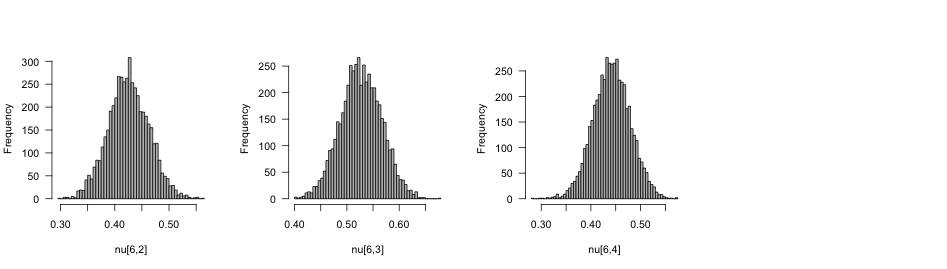

Supplement: S4 Appendix — (DOCX) [file pone.0117216.s004.docx]
